# *Notes from the Field*: Surveillance for *Candida auris* — Colombia, September 2016–May 2017

**DOI:** 10.15585/mmwr.mm6715a6

**Published:** 2018-04-20

**Authors:** Patricia Escandón, Diego H. Cáceres, Andres Espinosa-Bode, Sandra Rivera, Paige Armstrong, Snigdha Vallabhaneni, Elizabeth L. Berkow, Shawn R. Lockhart, Tom Chiller, Brendan R. Jackson, Carolina Duarte

**Affiliations:** ^1^Grupo de Microbiología, Instituto Nacional de Salud, Bogotá, Colombia; ^2^National Center for Emerging and Zoonotic Infectious Diseases, Office of Infectious Diseases, CDC; ^3^Oak Ridge Institute for Science and Education (ORISE), Oak Ridge, Tennessee; ^4^Division of Global Health Protection, Center for Global Health, CDC.

After a 2016 CDC alert describing infections caused by the multidrug-resistant fungus *Candida auris* (*1*), the Colombian Instituto Nacional de Salud (INS) queried the country’s WHONET^†^ database of invasive *Candida* isolates to detect previous *C. auris* infections. No *C. auris* isolates were identified during 2012–2016. However, *C. auris* is often misidentified as *Candida haemulonii* (*2*), a yeast that rarely causes invasive infections, and 75 *C. haemulonii* isolates were reported during May 2013–August 2016. These isolates came primarily from patients in intensive care units in the country’s north region, approximately 350–600 km (220–375 miles) from Maracaibo, Venezuela, where *C. auris* cases were first identified in 2012 (*3*). Of the 75 reported Colombian *C. haemulonii* isolates in WHONET, INS obtained 45 isolates from six medical institutions dating from February 2015 through August 2016, all of which were confirmed to be *C. auris* by matrix-assisted laser desorption ionization-time of flight (MALDI-TOF) mass spectrometry. Based on these findings, INS issued a national alert and mandated reporting of suspected isolates on August 30, 2016^§^ (*3**,**4*). In September 2016, a team from INS, CDC, and medical staff members from hospitals with documented *C. auris* cases investigated the 45 MALDI-TOF–confirmed *C. auris* cases identified before the INS alert. This investigation involved two hospitals in the north region and two in the central region. Cases were clustered within specific hospital units, and surveillance sampling demonstrated transmission in health care settings (INS and CDC, unpublished data, 2018).

After release of the Colombian clinical alert, INS received suspected *C. auris* isolates for confirmatory testing, and during September 2016–May 2017, an additional 78 *C. auris* cases were identified from 24 health care facilities in nine states, resulting in a total of 123 confirmed *C. auris* cases (Figure), more than half (54.5%) recovered from the northern coastal region (Atlántico, Bolívar, and Cesar). The median age of all patients was 36 years (interquartile range = 2–62 years), and 75 (61%) were male. Children aged 0–18 years accounted for 39 (32%) cases, including 23 (19%) in infants aged <1 year. The majority (68; 56%) of cases were reported from the northern region, and 30 (24%) were reported from the central region. Isolates were recovered from blood (74; 60%), urine (11; 9%), respiratory specimens (10; 8%), the gastrointestinal tract (7; 5%), and other body fluids and body sites (8; 7%). For 13 (11%) cases, no information was available about the source of the *C. auris* isolate.

**FIGURE:**
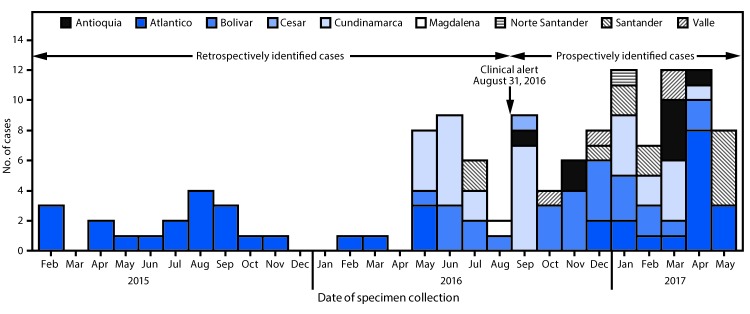
Confirmed cases of *Candida auris*, by month and state (n = 123) — Colombia, February 2015–May 2017

The VITEK 2 system had been used for yeast identification in 21 (75%) of 28 medical institutions. Four institutions used MicroScan (one), BD Phoenix (one), and Bruker MALDI-TOF Biotyper systems (two), and for three institutions, information about the identification method was not available. Six (4%) of 123 *C. auris* isolates were correctly identified, all by a clinical laboratory that used MALDI-TOF Biotyper (*2*). *C. auris* was most frequently misidentified as *C. haemulonii* (94; 76%), including 69 (97%) of 71 isolates identified by VITEK 2, all 23 isolates identified by BD Phoenix, and two of eight identified by MALDI-TOF Biotyper. Automated systems were unable to report a species for eight (7%) isolates (two by VITEK 2, four by MicroScan, and two by a system whose method was not reported). Thirteen *C. auris* isolates, all tested by MicroScan, were misidentified as other yeasts (*Candida albicans, Candida guilliermondii, Candida parapsilosis*, and *Rhodotorula rubra*).

Antifungal susceptibility testing was performed on 93 (76%) isolates^¶^ (*2**,**5*). Overall, 28 (30%) were resistant to fluconazole, 20 (22%) to amphotericin B, one (1%) to anidulafungin (an echinocandin), and one to both amphotericin B and anidulafungin.

Infections caused by *C. auris* are occurring in Colombia; the pathogen has been present in Columbia since at least 2015, and case counts are increasing. The number of reported cases likely does not reflect the true number of infected and colonized persons because of underreporting and underdiagnosis, as well as misdiagnosis as other yeast species (*6*). To contain the spread of *C. auris* in Colombia, INS updated the *C. auris* national clinical alert in July 2017 specifying which yeast isolates must be sent to INS for confirmation and mandating that medical facilities implement enhanced infection control practices, including using contact precautions and single rooms for patients with *C. auris* infections, minimizing the number of health care personnel in contact with infected patients, and daily and terminal cleaning of patient rooms and medical equipment with a disinfectant effective against *Clostridium difficile* spores** (*2*). Clinical laboratories should be aware that automated laboratory systems might incorrectly identify *C. auris*, particularly as *C. haemulonii*, although the species reported depends on the system (*2*).

## Footnotes

† WHONET is a free software program developed by the World Health Organization (WHO) Collaborating Centre for Surveillance of Antimicrobial Resistance to support national surveillance activities in more than 120 countries (http://www.whonet.org/index.html).

§
https://www.minsalud.gov.co/sites/rid/Lists/BibliotecaDigital/RIDE/IA/INS/ins-alerta-colombia-candida-auris.pdf .

¶ The broth microdilution method was used for azoles and echinocandins and Etest for amphotericin B; susceptibility breakpoints used were those described by CDC.

** http://www.famisanar.com.co/wp-content/uploads/documentos/POS/Men%C3%BA%20Sivigila/Circulares%202017/Infecciones%20Invasivas_%200025%20DE%202017%20INS%20CANDIDA.pdf.
